# Computational Paradigms for Mental Health

**DOI:** 10.1155/2017/5607631

**Published:** 2017-07-26

**Authors:** Pietro Cipresso, Aleksandar Matic, Guillaume Lopez, Silvia Serino

**Affiliations:** ^1^Applied Technology for Neuro-Psychology Lab, Istituto Auxologico Italiano, Milan, Italy; ^2^Department of Psychology, Università Cattolica del Sacro Cuore, Milan, Italy; ^3^Telefónica I&D, Barcelona, Spain; ^4^Aoyama Gakuin University, Kanagawa, Japan

Technology is allowing the acquisition of unprecedented information related to individuals' mental wellbeing as well as the provision of novel methods of treatment. The use of computational devices leverages the opportunities offered by the new research advancements to provide an improved diagnosis and a better support to both healthy subjects and patients but also to extend the theoretical knowledge.

Computational paradigms related to both new technologies and mathematical models have now many uses in psychology and cognitive science and can be a great opportunity for mental health research and clinical practice.

The experience of using computational tools is profoundly affected by the extent to which their use is easy, accessible, and above all accepted. In the last decades the use of computer-based assessment has hugely increased and we are now living the era in which technology is becoming accepted by both the practitioners and the patients. However technology needs to be defined within a paradigm and to be believable and then accepted. In this regard, we are soliciting high-quality papers able to capture the links between computational paradigms (related to technological tools, paradigms, models, simulation, and experiments) and mental health with regard to both wellness and illness.

This Special Issue has two foci, namely, to feature works that (a) advance scientific knowledge in the area of computational science applied to mental health and (b) explore deep investigation methods, techniques, and instruments to foster new ways to assess, assist, modulate, and understand the mental processes underlying behavioral, cognitive, and emotional aspects.

This Special Issue received several articles, accepting for publication five exciting contributions to the field.

E. Pedroli et al. developed a keen psychometric tool for a virtual reality rehabilitation approach to be used for dyslexia in children. According to the authors' results, an approach using cues in the context of a virtual environment may represent a promising tool to improve attentional skills. Virtual reality represents today one of the most interesting computational approaches to gather behavior and to deploy intervention in clinical and experimental settings. R. F. Navarro et al. explored the use and adoption of an assisted cognition system to support therapies for people with dementia. Evaluation results indicate that intervention personalization and a touch-based interface encouraged the adoption of the system, helping reduce challenging behaviors in patients with dementia and caregiver burden. The use of personalized ambient-assisted occupational therapies is gathering great interest in mental health field and the extensive use of low-cost technologies is increasing the stakeholders' attention. G. Marzinotto et al. explored the original approach for online handwriting style characterization based on a two-level clustering scheme exploring in particular age-related evolution patterns in online handwriting. Unlike previous works claiming that there is only one pattern of handwriting change with age, their study reveals three major aging handwriting styles, one specific to aged people and the two others shared by other age groups. The analysis of spatiotemporal handwriting parameters is an intriguing issue in computational analysis for mental health and became of huge attention in recent years. C. Romero-Rebollar et al. explored the neural modulation in aversive emotion processing. Independent Component Analysis (ICA) was carried out on a fMRI set obtained with a paradigm of face emotional processing. The results showed that an independent component, mainly cerebellar-medial-frontal, had a positive modulation associated with fear processing. ICA could serve as a method to understand complex cognitive processes and their underlying neural dynamics. In this sense, a computational approach has a key role in the further understanding of mental health process. C. Montag et al. provide insight into an emerging research discipline called psychoinformatics. In particular the authors provide a paradigm extension to understand the use of computer science in psychology under the umbrella of computational science, where mental health can be strongly investigated with advanced tools and methods. New challenges await psychologists in light of the resulting “Big Data” sets, because classic psychological methods will only in part be able to analyze this data derived from ubiquitous mobile devices, as well as other everyday technologies. As a consequence, psychologists must enrich their scientific methods through the inclusion of methods from informatics and computational science.

We hope that this Special Issue will foster a wider discussion for these exciting themes in computational paradigms for mental health.

*Pietro Cipresso* *Aleksandar Matic* *Guillaume Lopez* *Silvia Serino*

